# Renal Adenosarcoma Mimicking a Malignant Pelvocalyceal Tumor: An Interesting Imaging Case

**DOI:** 10.3390/diagnostics16030410

**Published:** 2026-01-28

**Authors:** Su Hong Kim, Hee Jung Kwon

**Affiliations:** 1 Department of Radiology, Yeungnam University Medical Center, Yeungnam University College of Medicine, Daegu 42415, Republic of Korea; drshkim@yu.ac.kr; 2 Department of Pathology, Yeungnam University Medical Center, Yeungnam University College of Medicine, Daegu 42415, Republic of Korea

**Keywords:** adenosarcoma, kidney, renal tumor, sarcoma, biphasic neoplasm

## Abstract

We report a rare case of primary renal adenosarcoma in a 26-year-old woman presenting with right flank pain. Contrast-enhanced computed tomography demonstrated a large, mixed solid and cystic mass confined to the renal pelvocalyceal system, closely mimicking a malignant renal tumor. Histopathologic examination revealed a biphasic tumor with phyllodiform architecture. Immunohistochemistry showed benign epithelial positivity for cytokeratin and focal malignant stromal positivity for smooth muscle actin and CD10; however, the tumor was negative for CD99, Wilm’s tumor protein, SS18-SSX, BCOR, and estrogen/progesterone receptors. These findings led to the diagnosis of primary renal adenosarcoma. This case highlights the diagnostic challenge of distinguishing this rare tumor from more common renal malignancies and underscores the importance of imaging–pathologic correlation.

**Figure 1:**
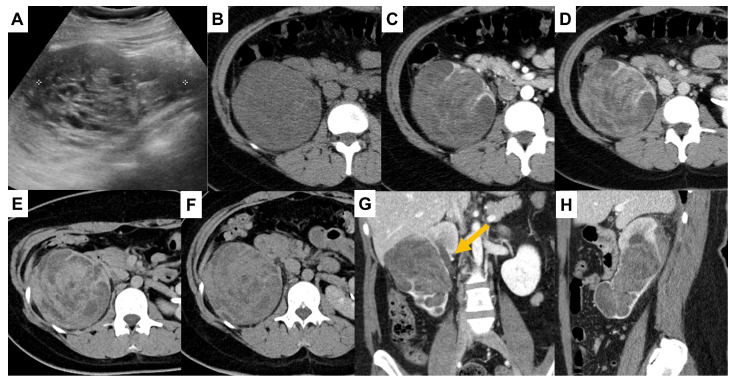
Primary renal adenosarcoma is an exceptionally rare biphasic tumor, particularly in young female patients, and its radiologic appearance may closely resemble more common malignant renal or urothelial neoplasms. Awareness of this entity is important, as misinterpretation based on imaging alone may lead to diagnostic pitfalls. The following images illustrate the key imaging and histopathologic features of this rare tumor and highlight the importance of imaging–pathologic correlation. A 26-year-old woman presented to our hospital with right flank pain. Her medical history and routine blood test analysis were unremarkable. Urine microscopy revealed numerous red blood cells and 30–40 white blood cells per high-power field. Additionally, a moderate amount of non-squamous epithelial cells and few squamous epithelial cells were identified. Ultrasonography demonstrates a 10 cm, partially cystic, heterogeneous echogenic mass in the right kidney (**A**). Contrast-enhanced renal CT images demonstrate a 14 cm × 10 cm × 7 cm hypodense mass on the non-contrast phase (**B**). The lesion exhibits mixed solid and cystic components with mild heterogeneous enhancement on the corticomedullary phase (**C**). The tumor demonstrated a gradual increase in attenuation from 27 HU on the non-contrast phase (**B**) to 43 HU on the corticomedullary phase (**C**) and 70 HU on the excretory and 20 min delayed phases (**D**,**E**), followed by a decrease to 50 HU on the 2 h delayed phase (**F**). This enhancement pattern differs from that of clear cell renal cell carcinoma, which typically demonstrates marked early enhancement on the corticomedullary phase (approximately 125 HU), and from urothelial carcinoma of the pelvocalyceal system, which shows moderate corticomedullary enhancement (approximately 91 HU) with relatively stable attenuation on excretory-phase imaging [1,2]. Coronal and sagittal excretory-phase images reveal that the mass is predominantly located in the interpolar region and lower pole, confined to the pelvocalyceal system, with associated pelvocalyceal dilatation and cortical thinning (**G**,**H**). The upper pole cortex is preserved and demonstrates a duplicated collecting system ((**G**), arrow).

**Figure 2:**
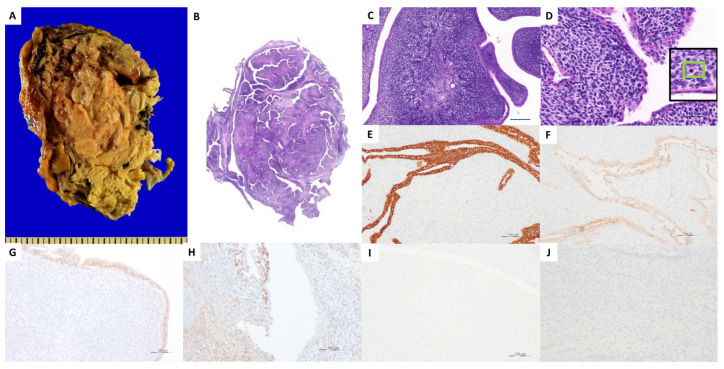
The patient underwent right nephrectomy due to radiologic suspicion of a malignant renal tumor. Gross examination reveals a 16 cm × 10 cm × 8 cm papillary mass within the renal pelvocalyceal system. The mass showed focal areas of hemorrhage with associated cortical thinning. No capsular, perinephric fat, or ureteral invasion was identified (**A**). The cut-surface of the tumor resembled a phyllodes tumor of the breast on low magnification ((**B**): HE, ×1). These cleft-like spaces are lined with bland cuboidal epithelium, and cellular stroma is located around the epithelium ((**C**): HE, ×40). In contrast to benign-appearing epithelial lining in tubules and cysts with flat, cuboidal, columnar, and hobnailed appearances, the highly cellular stroma component of the tumor shows periglandular condensation and cytological atypia. Epithelial–mesenchymal transition, carcinoma components, ovarian-type stroma, and blastemal elements were not identified. The atypical stromal cells have oval or spindly nuclei, and frequent mitotic activity is observed (up to 10 mitoses per 10 HPF) ((**D**): HE, ×200). Some of these cells show enlarged and more atypical/pleomorphic nuclei. Immunohistochemically, the epithelial components of the biphasic tumor are positive for cytokeratin (AE1/AE3) ((**E**), ×100), epithelial membrane antigen ((**F**), ×100) and paired box protein 8 (PAX8) ((**G**), ×100). The stromal cells are positive for smooth muscle actin ((**H**), ×100). The stromal cells show focal positivity for CD10. Ancillary immunohistochemical studies for desmin ((**I**), ×100), SS18-SSX ((**J**), ×100), TLE1, CD99, myogenin, estrogen receptor, progesterone receptor, BCOR, CD34, and pan-TRK are negative. Based on the biphasic morphology and immunophenotype, a diagnosis of primary renal adenosarcoma was established. Primary renal adenosarcoma is extremely rare, with only a limited number of cases reported in the literature [3]. Although adenosarcoma most commonly arises in the female genital tract, rare cases of primary renal adenosarcoma with comparable biphasic morphology and immunophenotypic features have been described [3]. The patient remained disease-free for 47 months of follow-up; however, subsequent clinical follow-up was not available because the patient was lost to follow-up. We considered the following differential diagnoses based on macroscopic and microscopic features. Primary renal sarcomas are rare and encompass a heterogeneous group of mesenchymal neoplasms, requiring careful morphologic and immunohistochemical evaluation for accurate diagnosis. Clear cell sarcoma of the kidney was also excluded due to the absence of characteristic arborizing fibrovascular septa and negative expression of TLE1 and BCOR. Additionally, Ewing sarcoma and embryonal rhabdomyosarcoma were excluded based on negative immunoreactivity for CD99 and myogenin, respectively. Synovial sarcoma (SS) is a distinct entity among primary renal sarcomas and represents a true biphasic tumor. Cystic change is common and is frequently associated with hobnail epithelium. However, in the present case, the epithelial component lacked the cytologic atypia characteristic of biphasic SS. Furthermore, SS18–SSX immunohistochemistry, which has recently been recognized as a useful surrogate marker for synovial sarcoma, was negative, along with CD34 and TLE1, making this diagnosis unlikely [4]. Congenital mesoblastic nephroma (CMN) is a rare renal tumor that most commonly occurs in infancy and is associated with an excellent prognosis. This diagnosis was excluded because CMN typically presents within the first year of life, whereas our patient was an adult, and pan-TRK immunohistochemistry was negative, arguing against an ETV6–NTRK3 fusion-associated tumor. Mixed epithelial and stromal tumor (MEST) is a rare renal neoplasm, and sarcomatous transformation has been reported in a limited number of cases [5]. Hobnail epithelial cells and spindled stromal cells resembling ovarian stroma are commonly observed in adult cystic nephroma (ACN) and MEST, and periglandular stromal condensation is a characteristic feature. Estrogenic influence and ectopic Müllerian remnants have been proposed as pathogenetic mechanisms, particularly in the setting of long-term hormone exposure and stromal overexpression of estrogen and progesterone receptors. In the present case, however, tumor cells were only focally positive for CD10 and were completely negative for estrogen and progesterone receptors, with no residual benign ACN or MEST component identified, arguing against this diagnosis. The large size of the lesion, the high cellularity and the phyllodiform architecture are extremely rare features in MEST. WT-1 negativity in stroma excludes the possibility that this is a metanephric adenosarcoma. The key immunohistochemical features of the present case and those of major biphasic renal tumors considered in the differential diagnosis are summarized in Table S1 (Supplementary Materials) [3,4,6,7,8,9,10]. To the best of our knowledge, there are no previous reports in the literature describing a primary biphasic tumor occurring in the kidney of a young female patient. The patient had no history of breast or gynecologic disease, had never undergone surgery related to these organs, and had no history of exogenous hormone exposure. Taken together, these clinicopathologic findings support the diagnosis of primary renal adenosarcoma. To date, only a single case adenosarcoma has been reported in the English literature by Sameshima et al., which occurred in an elderly female patient [3]. Although adenosarcoma most commonly arises in the female genital tract, a rare case of primary renal adenosarcoma with comparable biphasic morphology and immunophenotypic features has been described [3]. In the present case, we consider the epithelial component to represent a true neoplastic component rather than entrapped nonneoplastic renal epithelium. The epithelial elements were isolated from normal renal tubules, were neither atrophied nor compressed by increased luminal pressure, and were evenly distributed throughout the tumor, supporting their neoplastic nature. The periglandular condensation of sarcomatous cells resembled that observed in nephrogenic tumors or mixed Müllerian tumors. The epithelial component exhibited a leaflet-like architecture similar to Müllerian tumors, with cuboidal, columnar, and hobnail morphologies, as well as a multilocular cystic pattern reminiscent of mixed epithelial and stromal tumor. We believe that estrogen and progesterone played a minimal role in the tumorigenesis of this case, given the young age of the patient, the absence of hormone therapy, and the lack of estrogen and progesterone receptor expression on immunohistochemistry. In the previously reported case, low serum estrogen levels were presumed due to a history of bilateral oophorectomy. The patient remained disease-free for 47 months of follow-up. However, given the extremely limited data available on primary renal adenosarcoma, the biological behavior of this tumor remains uncertain, and careful long-term follow-up is recommended.

**Figure 3:**
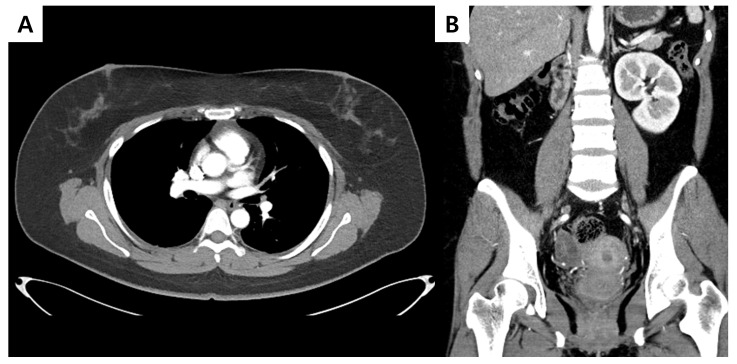
ChestCT obtained 3 months after surgery demonstrated no imaging findings suspicious for tumor recurrence or metastatic disease, including the bilateral breasts (**A**), and abdominal CT showed no evidence of another primary site or metastatic disease involving the gynecologic organs (**B**). Subsequent follow-up Chest and abdominal CT examinations performed at 13, 23, 35, and 47 months after surgery also revealed no evidence of recurrence or metastatic disease.

## Data Availability

Data sharing is not applicable (only appropriate if no new data is generated or the article describes entirely theoretical research).

## Supplementary Materials

The following supporting information can be downloaded at: https://www.mdpi.com/article/10.3390/diagnostics16030410/s1. Table S1: Summary of immunohistochemical findings distinguishing the present case of primary renal adenosarcoma from other biphasic renal neoplasms.

## Abbreviations

The following abbreviations are used in this manuscript:

CT	Computed tomography
HU	Hounsfield unit
HPF	High-power field
HE	Hematoxylin and eosin
PAX8	Paired box protein 8
TLE1	Transducin-like enhancer of split 1
BCOR	BCL6 corepressor
