# Supplementary material for: Renal Adenosarcoma Mimicking a Malignant Pelvocalyceal Tumor: An Interesting Imaging Case
Source: Diagnostics (Basel). 2026 Jan 28;16(3):410. doi: 10.3390/diagnostics16030410 (PMC12896551; doi:10.3390/diagnostics16030410)
Supplement: Supplementary file 1 [file diagnostics-16-00410-s001.zip › diagnostics-4118702-supplementary.pdf]

Table S1. Summary of immunohistochemical findings distinguishing the present case of primary renal adenosarcoma from other biphasic renal neoplasms.

Differential diagnosis	Adenosarcoma (current case)		Adenosarcoma [3]		MEST with sarcomatous change [6, 7]		Metanephric Adenosarcoma [8, 9]		Synovial sarcoma (Biphasic) [4, 10]	
	Epithelial cells	Stromal cells	Epithelial cells	Stromal cells	Epithelial cells	Stromal cells	Epithelial cells	Stromal cells	Epithelial cells	Stromal cells
**CK(AE1/AE3)**	+	-	+	-	+	-	+	-	+	-
**EMA**	+	-	+	-	+	-	+	-	+	-
**Desmin**	-	-	-	-	-	+	-	-	-	-
**SMA**	-	+, some	-	-	-	+	-	-	-	+, focal
**Myogenin**	-	-	-	-	-	variable	-	-	-	-
**WT1**	-	-	-	-	-	+/-	-/+	+	-	-
**S100**	-	-	-	-	-	-/+	NA	-	-	-/+
**TLE1**	-	-	+	+	-	-	NA	NA	-	+
**ER**	-	-	-	-	-	+/-	NA	NA	-	-/+
**PR**	-	-	-	-	-	+	NA	NA	-	-
**CD10**	-	+, focal	-	+, partly	-	+	-	+	-	+
**CD34**	-	-	-	+, partly	-	-	NA	NA	-	-
**CD99**	-	-	-	+	-	+/-	NA	-	-	+
**SS18-SSX**	-	-	NA	NA	NA	NA	NA	NA	-	+<br>(fusion)
**BCOR**	-	-	NA	NA	NA	NA	NA	NA	NA	NA
**Pan-TRK**	-	-	NA	NA	NA	NA	NA	NA	-	-
**PAX8**	+, focal	-	NA	NA	+	-	NA	NA	+/-	-
**Histologic features**	Phyllodiform architecture		Phyllodiform architecture		Ovarian-type stroma		WT1-positive stroma		True biphasic	
